# Real Time Analysis of Bioanalytes in Healthcare, Food, Zoology and Botany

**DOI:** 10.3390/s18010005

**Published:** 2017-12-21

**Authors:** Tianqi Wang, Ashwin Ramnarayanan, Huanyu Cheng

**Affiliations:** 1 Department of Engineering Science and Mechanics, The Pennsylvania State University, University Park, PA 16802, USA; wtqwad123@gmail.com; 2 School of Engineering Design, Technology and Professional Programs, The Pennsylvania State University, University Park, PA 16802, USA; azr5383@psu.edu; 3 Materials Research Institute, The Pennsylvania State University, University Park, PA 16802, USA

**Keywords:** bioanalytes, wearable technology, biosensors, healthcare, food, zoology, botany

## Abstract

The growing demand for real time analysis of bioanalytes has spurred development in the field of wearable technology to offer non-invasive data collection at a low cost. The manufacturing processes for creating these sensing systems vary significantly by the material used, the type of sensors needed and the subject of study as well. The methods predominantly involve stretchable electronic sensors to monitor targets and transmit data mainly through flexible wires or short-range wireless communication devices. Capable of conformal contact, the application of wearable technology goes beyond the healthcare to fields of food, zoology and botany. With a brief review of wearable technology and its applications to various fields, we believe this mini review would be of interest to the reader in broad fields of materials, sensor development and areas where wearable sensors can provide data that are not available elsewhere.

## 1. Introduction

Chemicals, physical, or biological reaction products, i.e., biomarkers, produced from a bodily function within the organism can be detected by sensors either invasively or non-invasively to form the basis of bioanalysis. The World Health Organization has defined a biomarker as “almost any measurement reflecting an interaction between a biological system and a potential hazard, which may be chemical, physical, or biological. The measured response may be functional and physiological, biochemical at the cellular level, or a molecular interaction” [1].

Physical biomarkers range from biopotential signals (e.g., electroencephalogram, EEG; electrocardiogram, ECG; electromyogram, EMG) to vital signs such as body temperature, pulse rate, respiration rate and blood pressure [2]. Routine measurements of these vital signs provide critically important information for the body’s most basic functions. Reviews on wearable physical sensors are both numerous and overwhelmingly positive [3,4,5,6], we will focus on wearable chemical/biological sensors (referred as biosensors in the following discussion) in this mini review. Such a wearable biosensor measures chemical or biological reactions by generating signals proportional to the concentration of an analyte in the reaction. The receptor with biological (e.g., enzymes, aptamers, antibodies, DNA) [7] or chemical components [8,9] in the biosensor interacts with the analyte to generate output signals (in the form of light, heat, pH, or others) with a defined sensitivity. The output signals are either measured directly or converted by the transducer into electrical signal for easy quantification.

Compared to complex and high cost analytical instruments for intermittent measurements, wearable biosensors provide an alternative to address such a challenge by continuously monitoring biomarkers/analytes [1]. In past decades, the successful realization of wearable sensing technology enabled the migration of electrochemical systems from traditional rigid and planar substrates to flexible and stretchable bases [3,10]. The enabled in situ monitoring platforms can maintain good performance under repeated bending and tension from normal on-body wearing. Such wearable platforms are critical for a wide range of fields as they provide a continuous monitoring that could not be achieved by traditional wafer-based technologies [3,11,12]. The benefit of real time monitoring is not reserved to people with ailments but also to healthy individuals (e.g., performance status of athletes) [13].

However, it should be noted that biomarker testing could only be used as a surrogate endpoint but not as a clinical endpoint in most cases. As per the Food and Drug Administration, a surrogate endpoint is “a laboratory measure or a physical sign that is intended to be used as a substitute for a clinically meaningful endpoint” [14]. A representative example of this is body temperature, a physical biomarker. A high temperature can be interpreted as a symptom for an underlying condition but may not be used as the diagnosis all by itself. Continuous monitoring of the body temperature over time will help medical professionals narrow down the list of possible diseases and order relevant clinical testing to provide the clinical endpoint needed for the diagnosis. Therefore, it fuels the demand for wearable devices that can continuously monitor the biomarkers in real time.

In this mini review, we will first introduce the stretchable materials and structures for the wearable sensors and discuss the manufacturing techniques of various wearable sensors in Section 2. Next, we will highlight the enabled applications from healthcare in Section 3 to the fields of food, zoology and botany in Section 4. We will then provide our perspective on the future direction of research development and conclude the mini review in Section 5. It is worth mentioning that with the development of wireless sensor networks (WSNs) and the Internet of Things, individual in situ monitoring devices could connect to an integrated network for intelligent control and data analysis [15]. But in this mini review, we mainly focus on wearable sensors technologies that monitor biomarkers and obtain specific information from the subject.

## 2. Material, Structure and Fabrication of Wearable Sensors

The stretchable materials and structures are indispensable components in constructing stretchable and wearable sensing devices. Meanwhile, the advanced manufacturing technology has significantly cut the cost and enabled customized production of wearable sensors, paving the way for potential commercialization. Considering the numerous excellent reviews in this field [3,16,17,18,19], we will briefly discuss the recent development of stretchable materials and structures and then manufacturing techniques for wearable biosensors in this section.

### 2.1. Stretchable Materials and Structures

**Figure 1:**
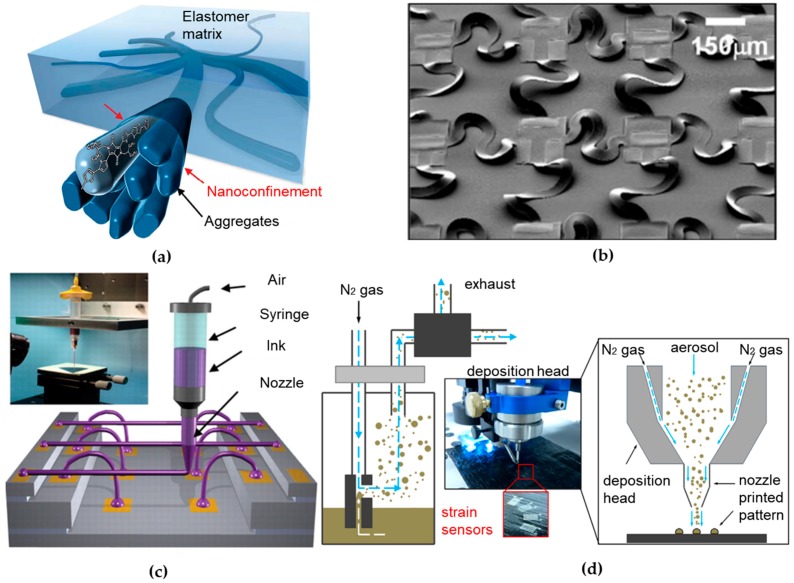
Material, structure and fabrication of wearable sensors. (**a**) A 3D schematic of the polymer semiconductor with embedded nanoscale networks to achieve high stretchability. Reprinted with permission from [27]. (**b**) SEM mage of an array of stretchable complementary metal-oxide-semi-conductor inverters with noncoplanar bridges that have serpentine layouts. Reproduced with permission from [34], Copyright (2008) National Academy of Sciences. (**c**) Schematic diagram of omnidirectional direct ink writing (DIW) printing setup and optical image of apparatus is shown in the inset. In such setup, concentrated ink is dispensed through a tapered cylindrical nozzle that is translated using a three-axis, motion-controlled stage with microscale precision. Reprinted with permission from [35]. (**d**) Schematic of the functional principle of the aerosol jet printing (AJP) system. The silver ink is first aerosolized to form a gas stream and transported to a print head. It is then focused in a sheath gas flow and sprayed toward the substrate to print strain sensors. Reprinted with permission from [36].

The fundamental mismatch in materials and geometries between soft biological tissues and traditional rigid electronics hinders the development of devices capable of intimate, conformal integration with biological tissues. The concept of flexible and stretchable electronics was conceived to bridge the fundamental mismatch. The capability of a given material to flex is determined by its bending stiffness that scales with the cubic of its thickness, indicating a thin geometry for flexibility. The development of flexible electronics started around half century ago, when the single crystal silicon wafer was thinned to ~100 μm and assembled on a plastic substrate to build the first flexible solar cell array [20]. Stretchable electronics can be achieved either by using new stretchable materials in conventional layouts, or by exploiting new structural layouts with conventional materials. Organic conducting and semiconducting polymers are initially developed for their light weight and intrinsic flexibility to withstand large deformation [21]. Since the invention of conductive polyacetylene in the 1970s, conducting polymers have received significant attention for their good electrical and optical properties, while maintaining mechanical flexibility and processability of polymers [22]. By either binding additional molecules in the conjugated structure [23,24] or dispensing advanced nanomaterials or nanostructure [25,26,27] in the stretchable polymers (Figure 1a), novel conducting/semiconducting polymer can be easily optimized for a specific application. In addition, the charge carrier mobility of semiconducting polymers has reached or exceeded the value of amorphous silicon [28]. With excellent mechanical properties and ease of manufacturing from conventional solution processing technologies, a wide variety of electrical and optical devices have been demonstrated using semiconducting polymers, including polymer light-emitting diodes (PLEDs) [29], organic thin film transistors (OTFTs) [30], organic photovoltaic cells (OPVs) [31] and electronic sensors [32]. However, conducting/semiconducting polymers are still associated with poor stability and compromised electrical performance (e.g., low conductivity or charge carrier mobility), in comparison to their inorganic counterparts [33]. 

Another branch of stretchable electronics that is based on well-established inorganic materials in the industry has been well devolved since mid-2000 [37]. In such effort, intrinsically stiff and brittle inorganic electronic materials are fabricated in a thin geometry, followed by heterogeneous integration [38,39] on flexible sheets to create circuits that can bend and flex much like organics. A variety of novel structural designs have been created for stretchable circuits, in which wavy geometry is a key feature [40]. One representative design configures thin films of inorganic materials into “wavy” shapes, which are then fully bonded [41,42] or selectively bonded [43] to elastomeric substrates [44,45,46,47,48,49]. The system can sustain external deformations by changing the wavelength and amplitude of the wavy shapes. Another relatively more advanced structural design involves inorganic materials patterned into isolated micro-islands and serpentine-shaped interconnect ribbons between islands, typically termed as the “island-bridge” design shown in Figure 1b [34,50,51,52,53]. Other advanced structural designs involve exploitation of fractal [54,55,56,57], origami [58,59,60,61] and many others [62,63,64]. The stretchable structure removes the limitation of the stretchable characteristics in the device materials to enable the use of conventional and advanced materials, including metals (nanoparticles [65,66], nanowires [67,68]), inorganic semiconductors (nanotube, nanomesh, quantum dots [69,70,71]) and low dimensional materials (single-walled carbon nanotubes (SWNTs) [72,73], graphene [74]). These innovative structures and emerging materials provide stretchable/flexible electronic systems with circuit performance characteristics comparable to on-wafer/chip-level counterparts. Capable of overcoming the aforementioned fundamental mismatch, ultrathin tattoo-like electronic devices [6,75] have been developed to directly interface with human body for non-invasive, real-time and continuous monitoring of physiological signals (e.g., temperature [76,77], strain [78,79,80] and electroencephalogram/electrocardiogram/electromyogram [81,82,83,84]) to inform health status [85]. When used in implantable devices [86,87], it is also of great interest to explore biodegradable materials [88,89,90,91,92,93,94] towards a physically transient form of stretchable electronics [95].

### 2.2. Manufacturing Techniques

Fabrication methods play an important role in determining the performance, cost and stability of stretchable/wearable electronics. While various manufacturing technologies have been developed for fabricating wearable sensors in past decades, lithographic processes and printing methods are two main technologies among them [96]. As a mature manufacturing technology, lithographic processes (e.g., deposition, photolithography, etching) could fabricate high resolution wearable electronics with performance comparable to on-wafer counterparts [97]. The lithographic processes with a highly controlled deposition and patterning capability transfer biological components onto substrate in the precise defined location to interact with analyte in a sample fluid. For example, soft lithography is used to define sealed microfluidic channels and reservoirs with soft polymers (e.g., polydimethylsiloxane, PDMS) in a wearable sweat sensor for sweat collection and analyte analysis (see Section 3.1 for more details) [98]. Such standard soft lithographic techniques have also enabled fabrication of epidermal sensing devices [99,100]. However, the lithographic techniques are costly due to their complicated fabrication steps, requirement of cleanroom environment and labor-intensive processes in the research labs.

As an alternative approach, printing techniques are simple, rapid, low cost and efficient for customized production with desired precision and accuracy. The current technologies of printing electronics can be broadly classified into template and non-template based methodologies [96]. Template-based printing processes include screen printing, gravure printing, flexography and many others [101]. As a conventional technique for film deposition and patterning of both organic and inorganic materials [102], screen printing is one of the most widely used techniques in the manufacture of biosensors (e.g., half of the disposable glucose sensors are fabricated by screen printing) [103]. During the screen-printing process, a liquid paste is forced through a patterned mesh/stencil mask by a rubber squeegee to form a pattern onto the substrate surface. As to be discussed more in details in Section 3.1, wearable biosensors have been screen printed on polyethylene terephthalate (PET) stickers, followed by placement on each side of the glasses’ nose pads in an integrated eyeglass sensing platform to monitor sweat metabolites and electrolytes [104]. Capable of transferring the ink materials to form patterns on the substrate from a roller, gravure and flexography can be extremely useful for high throughput production of large-area flexible/stretchable devices.

Non-template printing techniques rely on localized and controlled ink dispensing onto the receiving substrate. Direct ink writing (DIW) technologies, in a broad definition, are being rapidly developed over the past decades for non-template printing. These technologies basically employ a computer-controlled translation stage and a pattern generating device (e.g., ink-deposition nozzle) to create patterns with a specific material composition and structural architecture (Figure 1c) [19]. DIW techniques are classified into two sub-groups: droplet-based and extrusion-based systems [105]. Inkjet printing and aerosol jet printing (AJP) are two representative techniques in the former group. Inkjet printing allows deposition of small volumes (~1–30 pL) of ink onto a small, pre-determined area with high precision (line width resolution of ~40 μm) [106]. In the example in Section 3.1, an array of silver interdigitated electrodes coated with a stretchable sensing film in a wearable e-nose has been inkjet-printed to monitor the axillary odor released from human body [107]. In the AJP system, the functional ink is first aerosolized to form a gas stream and transported to a print head, followed by focusing in a sheath gas flow and spraying toward the substrate (Figure 1d) [108]. Capable of printing various materials that include metals [109], polymers [110], ceramics [111] and even biological materials [112], the AJP system can create complex 3D patterns on both planar and non-planar substrates with a very high resolution (~10 μm) [113]. These capabilities make it suitable to fabricate a wide range of highly customizable printed electronic devices, such as thin film transistors [114,115], printed circuit boards [116,117] and biosensors [112,118,119]. Extrusion-based system has the same working principle barring that they deposited material in a continuous flow. When coupled with a focused laser [120] or ultraviolet illumination [121], freestanding 3D metallic or polymeric architectures at the microscale can also be directly printed.

Moreover, novel manufacturing techniques are emerging with new materials and/or designs for wearable sensors, such as fiber/textile based electronic devices that exploit traditional textile processing technologies [122]. Another representative example of “do it yourself” biosensor allows enzymatic-ink-based roller pens to directly draw renewable glucose sensor strips on human skin and various unconventional surfaces with minimal user training [123]. As wearable sensing devices are relatively complex devices due to its diverse components, the combination of different manufacturing methods in an efficient manner may be one important direction of future developments.

## 3. Biosensors in Healthcare

Biomarker analysis for healthcare is in the forefront of development. Medical professionals seek sensing systems that are able to gather vital information from the body without the need for expensive invasive procedures [124,125,126]. In addition to physical signals, measurement of chemical or biological substances allows medical professionals to predict the onset of a disease and measure the progression of the said disease. Of particular interest to the non-invasive detection and quantification, a majority of the chemical or biological substances are present in bodily fluids (e.g., sweat, saliva and tears) or even the body odor [127] (Table 1).

### 3.1. Wearable Biosensors

**Figure 2:**
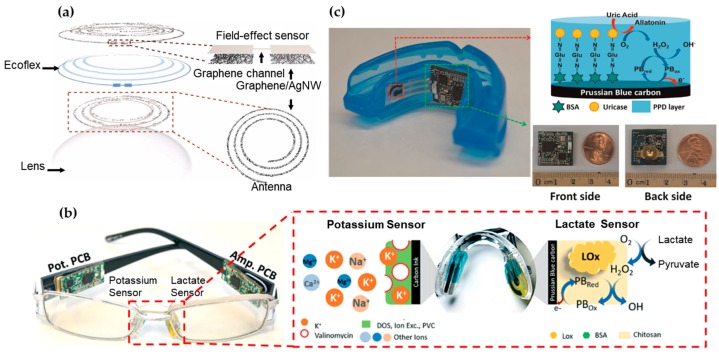
Wearable sensors for biomarker detection in tear, saliva and their odor. (**a**) Schematic of the wearable contact lens sensor, integrating the glucose sensor and intraocular pressure sensor. The graphene-silver nanowire (AgNW) hybrid structure has enhanced electrical and mechanical properties while maintaining high transparency. Reprinted with permission from [131]). (**b**) Nose pad electrochemical sensors with potassium sensor (**Left**) and lactate sensor (**Right**), along with the corresponding recognition and transduction events. Reprinted with permission from [104]. (**c**) Photograph of the mouth guard biosensor integrated with wireless amperometric circuit board (**Left**). Reagent layer of the chemically modified printed Prussian-Bluecarbon working electrode containing uricase for salivary uric acid biosensor (**Top Right**). Photograph of the wireless amperometric circuit board (**Bottom Right**). Adapted with permission from [130].

Containing water, glucose, lactate, proteins and many other biomarkers [132], tear is a representative bodily fluid for biomarker analysis. Certain tear proteins have been evaluated as indicators for ocular and systemic diseases, including glaucoma [133], diabetes mellitus [134] and cancer [134]. However, the current focus of wearable biosensors remains on monitoring of glucose and lactate concentrations in the tear [132]. Studies have shown a direct correlation between blood and tear glucose levels [135,136]. Tear sensors are most commonly attached to the inner side of the flexible contact lens for direct glucose monitoring in the iris (Figure 2a) [131,137,138]. The current challenge is the integration of an adequate power source for the sensor. The available surface area for the device is relatively low, because the sensor is always placed on the outskirts of the lens with minimum interference of the vision [139]. This has spurred the development of biofuel cells that employ enzyme catalysts to convert the glucose and oxygen into electrical energy for energy supply [140]. This concept can also be applied to the sensor design for the other biofluids due to the wide presence of glucose. When integrated with an on-glass radio-frequency identification (RFID) reader, an ultra-high frequency (UHF) RFID wireless transceiver can also be used for energy transmission [141]. Figure 2a presents a field-effect transistor (FET) with graphene as a channel to detect the glucose concentration from the change of drain current (or concentration of charge carriers in the channel), which results from the oxidation of glucose to gluconic acid catalyzed on the graphene channel. 

When implemented in a passive RLC circuit design, the resistance of the FET glucose sensor changes upon glucose binding, while the inductance (L) and capacitance (C) are increased for an intraocular pressure. An inductively coupled reader antenna allows simultaneously measurement of the glucose level in tear fluid (from reflection value at the resonant frequency) and intraocular pressure (from resonant frequency) [131]. Another representative example exploits a flexible oxygen sensor to measure transcutaneous oxygen tension from conjunctiva [142]. The conjunctiva has high gas penetration and supplies the cornea with oxygen, obviating the need for heating that could lead to skin burn. Demonstration on a conjunctiva of a Japanese white rabbit has shown increased sensor output in a high oxygen concentration in comparison to the standard air inhaling. Such sensor could be critically important to analyze arterial oxygen pressure in infants. It is essential to prevent oxygen poisoning caused by retinopathy of prematurity or to prevent hypoxia in neonatal intensive care units.

Eyeglasses represent another attractive wearable platform for wearable sensors. According to the Vision Council of America, approximately 64% of adults in the USA wear eyeglasses for vision correction or even as stylish fashion accessories such as Google Glass. As shown in Figure 2b, a wearable chemical sensing platform includes a potentiometric sensor for potassium measurement and an amperometric sensor for lactate measurement on two nose-bridge pads of glasses, as well as a wireless electronic backbone along the glasses’ arms [104]. The lactate biosensor relies on the amperometric measurement of the peroxide product from the LOx biocatalytic oxidation of lactate and the potentiometric sensor measures potassium concentrations through open-circuit potential. Each connected PCB is measured to consume an average current of 1.6 mA during operation, enabling a battery lifetime of over one week with 100 mA h capacitance (assuming 8 hours use per day). Due to well-developed electrochemical measurements of analytes, a variety of different amperometric and potentiometric sensor stickers can be applied on bridge-pad for other bioanalytes such as glucose.

The wireless biosensor integrated on a mouth guard can also monitor salivary uric acid (SUA) levels and even an assortment of other analytes for saliva testing (Figure 2c) [130]. The integrated amperometric sensor was used to offer a high sensitivity, selectivity and stability towards SUA detection, covering the concentration ranges for both healthy people and hyperuricemia patients. Unlike RFID-based biosensing systems that require large proximal power sources, the electrochemical sensor houses a Bluetooth low energy communication chip powered by two watch batteries of 1.55 V and 33 mAh in series. The board consumed 7 mA from a 3 V supply during the active mode (21 mW) and 0.6 mW in sleep mode, setting the battery life to ca. 5 days. However, the sustained presence of batteries in the mouth cavity incurs health risks that are unfavorable and hence the current focus is to develop a replacement for power supply.

Biomarkers are not limited to the biofluids upon their formation. Instead, they also include the odors upon reacting biofluids with other chemical or environmental factors. To detect the odor, a wearable e-nose has been developed to track real-time health status or body hygiene [107]. Due to its proximity to the armpit, the odor sensor worn around the arm can pick up volatile organic compounds (VOCs) such as ammonia, amines, hydrocarbons, alcohols, acids, ketones and aldehydes, which contain information about human health (inner factor) and skin hygiene (outer factor). With the volatiles penetrating the sub-surface of the carbon nanotubes (CNTs)/polymer film, the swelling of polymer would alter the conducting pathways of CNTs. The resulting change in the electrical resistance of the sensor has been recorded as a function of the presented VOCs and their concentrations. It can also be applied to detect odor stemming from perspiration, exhaled breath, or human excreta, allowing indirect measurement of biomarkers from multiple locations.

### 3.2. Tattoo-Like Biosensors

As a representative biofluid, sweat contains both electrolytes (e.g., sodium, potassium, calcium, magnesium, zinc) and biological markers (e.g., lactate, glucose, chloride, urea) [143] for healthcare monitoring. For example, the concentration change of electrolytes such as sodium and potassium in the sweat indicates tissue dehydration, muscle heat cramps and cardiovascular diseases [144,145,146]. The level of lactate can also reflect the oxidative metabolism of tissues corresponding to the exercise intensity [147]. Thus, the application of sweat analysis includes disease diagnosis [148], drugs and ethanol detection [149] and athletic healthcare [150].

**Figure 3:**
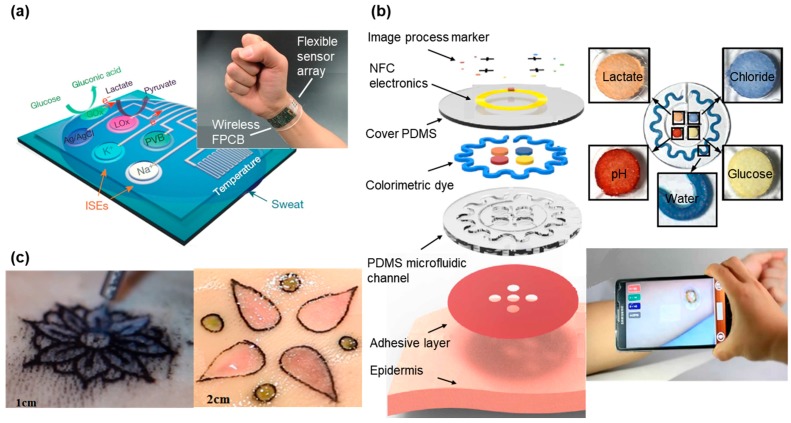
Tattoo-like sensor for biomarker detection. (**a**) Image and schematic illustration of the flexible integrated sensing array (FISA) for multiplexed perspiration analysis. Photograph of a wearable FISA on a subject’s wrist. The system consists of a multiplexed sweat sensor array and a wireless flexible printed circuit board (FPCB) (**Left**). Schematic of the sensor array that includes glucose, lactate, sodium, potassium and temperature sensors for multiplexed perspiration analysis is shown on the right. GOx and Lox: glucose oxidase and lactate oxidase. Adapted with permission from [128]. (**b**) Schematic illustration of an epidermal microfluidic sweat monitoring device. Colorimetric detection reservoirs enable determination of total water (sweat) loss and concentrations of lactate, glucose, creatinine, pH and chloride ions in sweat. Reprinted with permission from [98]. (**c**) Designs made by a tattoo artist in ex vivo pig skin. Tattoo artist designing with tattoo ink and chromogenic pH (**Left**) with chromogenic pH and glucose biosensors shown on the right. Reproduced from the video released in MIT DermalAbyss Project, permission from Creative Commons Attribution-Noncommercial 4.0 International.

Continuous detection of the multiple biomarkers is highly desirable for health monitoring but sweat-based biosensors typically focus on a single analyte at a time. In addition, lack of on-site signal processing circuitry and sensor calibration mechanisms makes them difficult for real-time, accurate analysis of the physiological state [14,15]. Figure 3a presents a fully integrated sensor array for multiplexed, in situ perspiration analysis, which simultaneously measures glucose and lactate (measured from chronoamperometric responses) and sodium and potassium ions (measured from open circuit potential responses), as well as the skin temperature [128]. The sensor array transforms the analyte concentration into electrical signals, which are processed by the flexible circuit board (FPCB) and then wirelessly transmitted to a smartphone via Bluetooth. The power is from a single rechargeable lithium-ion polymer battery with a nominal voltage of 3.7 V of a desired capacity. The microcontroller in the system is compatible with the popular Arduino development environment, featuring low power and low cost. The integrated system provides continuous monitoring from all the sensors, with excellent stability over the exercise period of a few hours. In particular, the sensor array could be repeatedly used for continuous temperature and sweat electrolyte monitoring. However, the responses from glucose and lactate sensors degraded beyond the exercise period owing to decreased enzyme activity. This could be easily addressed by convenient replacement of the fresh sensor arrays for subsequent use. Although capable of being worn on various body parts during exercise, this sensing platform still could be prohibitive for certain applications due to the size and cost of the FPCB up to date. As an alternative, Figure 3b presents a design with lightweight, low-cost and fashion considerations. In addition to the detection of biomarkers such as pH, lactate, chloride and glucose, this wearable sweat sensor features sweet rate analysis [98], which is related to thermal regulation and dehydration [150,151]. Upon intimate contact with the skin, the soft patch routes the sweat to different microfluidic channels and reservoirs. The serpentine channel with inside surface coated with a layer of CoCl_2_ provides an intuitive recognition of the real-time sweat rate, because the CoCl_2_ chelated with water forms CoCl_2_·6H_2_O to generate a change in color from deep blue to pale purple. Additionally, another four colorimetric chemical assays resided in the separate reservoirs interact with analytes in sweat for the detection of biomarkers. Instead of converting these signals to electric signals through transducer for further processing, the color change can be directly quantified. The colorimetric indicators can either be observed directly by eyes or be processed by image capture and analysis software in a smartphone to obtain a quantitative analyze of collected sweat. Compared with electrochemical biosensors that need electronic circuits and complicated transduction mechanisms [16,20,21], the optical biosensors are more practical and cheaper for rapid diagnostics in medicine and healthcare sectors [22]. However, it should be noted that the sensitivity of these colorimetric sensors might not be as high as those transduced electronic signals. Due to scant colorimetric reagents stored in the sensors, the outdoor exercise test is limited to a certain time frame. Although this tattoo-like sweat sensor is designed for one-time used, the stability of the sensor still needs to be further optimized to account for vapor loss and its limited capacity.

A similar idea is also behind the Dermal Abyss (d-abyss) project from the MIT media lab and Harvard Medical School started in 2017 (Figure 3c). Replacing traditional tattoo inks with colorimetric and fluorescent sensors, the researchers injected these biosensors below the skin to reflect inner metabolic processes from the concentration of sodium, glucose and pH in the interstitial fluid (ISF) [129]. As the main component of the extracellular fluid, the ISF surrounds the cells and contains various biomarkers and electrolytes [152]. The optical biosensor changes its color according to the different concentrations of analyte (sodium, potassium, pH and glucose) in the ISF. In addition, the aesthetic design in the tattoo makes the d-abyss highly attractive and desirable in health monitoring and cosmetics. While early in the research development for the Dermal Abyss, its stability, reversibility and range of detection could be improved in the future.

## 4. Application in Food, Zoology and Botany

Although the central focus of the current development of wearable sensors has been on healthcare, the common sensing principle and measurement signals also allow wearable technology to find applications in the fields of food, zoology and botany. The increasing interest has spurred the development of wearable sensing technology for food quality tracking, animal health monitoring and plants status detection.

### 4.1. Food Sensors

Some of the biomarkers tested in human beings can also be found in food therefore bioanalysis techniques can be applicable to food as well [153]. Because the consumption of the food provides nutrients for the human body, the monitoring of food quality is of increasing concern to the public. Most countries have a federal organization such as the Food and Drug Administration (FDA) in the United States of America that oversees food safety. The current focus remains on monitoring food quality in perishable food goods such as dairy and meat products. In order to detect food spoilage, there are a variety of accurate measurement methods, including gas chromatography [154], spectroscopy [155] and ultrasound interrogation [156]. The biosensor systems are also developed to test for chemicals that indicate spoilage. However, regardless of the high accuracy of these systems, they are neither cost effective nor user friendly for consumers to directly monitor the food quality at the point of consumption [157].

In order to address this challenge, researchers have attempted to create flexible sensing systems that do not contaminate the food but are still able to give relatively accurate results. One important type of sensors relies on the detection of changing dielectric values in the food over time, due to their indications of chemical changes of the food. In the example of milk, the spoilage results from the growth of microorganisms in the medium. The growth processes cause changes in the dielectric properties, viscosity and conductivity of the milk [158]. To measure the change in the dielectric constant, a radio frequency identification (RFID) antenna has been developed to sense the electric field change from the ambient environment (e.g., milk) [159]. Eliminating the need for a separate power source, the RFID tag can be placed on the outside surface of the milk container to reduce the risk of contamination. Such simple design also reduces the size of the sensor. When combined with inductor and capacitor, the RFID tag printed on a paper substrate can monitor humidity levels in a packaged product, which directly correlates with the food quality [160]. The water vapor absorbed by the paper substrate alters the resonant frequency through the change of the capacitance [161]. The single tag can also be integrated into a high-density sensor array with each to detect a specific analyte, improving the sensing fidelity, due to monitoring of multiple analytes that are pertinent to the perishable good. The array may even open up the opportunity to mimic the function of the nose (e.g., electronic noses [162]).

**Figure 4:**
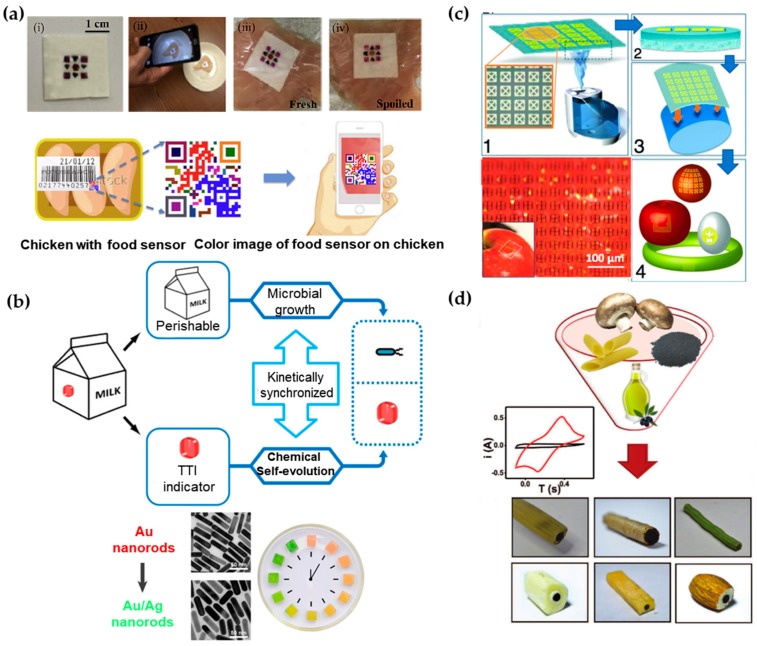
Various demonstrations of biosensors in the food industry. (**a**) Geometric barcode sensor for monitoring chicken spoilage under different temperature conditions (top): (i) image of fabricated sensor; (ii) image of smartphone-based detection; sensor placed on (iii) fresh and (iv) spoiled chicken at room temperature. The bottom shows schematic of device application as food quality sensor. The sensor can be attached to the surface of the meat or be placed onto the inside lining of the package. The status of the meat product can be monitored using smart phone by taking a photo of the sensor. Reprinted with permission from [157]. (**b**) Schematic illustrating the expected “biochemo” synchronicity. Microbial growth in the perishable product and the chemical chronochromic self-evolution of the time temperature indicator (TTI) are always kinetically synchronized, regardless of the temperature history. Bottom left shows chronochromic evolution from Au nanorods to Au/Ag nanorods. Bottom right is a photograph of the hydrogel cubes illustrating the red-to-green chronochromic performance. Reprinted with permission from [164]. (**c**) Schematic of steps for rapid transfer of silk antennas onto curved substrates: (1) Water vapor is applied to the back of silk films, yielding (2) a film in which the back surface of the film has been partially melted. (3) This melted surface is conformally applied to arbitrary surfaces, yielding (4) applied functional sensors on a variety of surfaces. Photos of THz split ring resonators (SRRs) fabricated on the silk substrate wrapped on an apple. Reprinted with permission from [156]. (**d**) Illustration of edible electrodes that are comprised of edible activated charcoal, edible food sleeves and olive oil loaded into different food products. Reprinted with permissions from [165].

As an alternative to direct readout of the information from the sensor with laboratory equipment or smartphones, colorimetric sensing array arranged in a barcode/QR code can be processed by a mobile phone camera [157]. In this sensor, a low-cost paper substrate is impregnated with cross-reactive vapor sensitive dyes encapsulated in resin microbeads (Figure 4a). When placed on chicken meat under different temperatures, the colorimetric dyes can respond to the VOCs emanated from the meat. The patterned barcode then allows the consumers to know the information of meat quality, by using their smartphones with a built-in app to process the colorimetric signal, similar to the tattoo sweat sensor discussed in Section 3.2. In addition to the meat spoilage demonstrated in this example, gas products of different food can be detected by incorporating other optical dyes such as pH indicators or Lewis acid [163] in this platform.

In certain applications, it is not straightforward to directly measure the property change for food spoilage. As an alternative, other factors (e.g., air, light and temperature) that would affect food quality during production, transportation and storage are measured [166]. For example, time temperature indicators (TTIs) that show the accumulated time-temperature history of a product as labeled on the package have been widely used in food and medical products [167,168]. Instead of direct measurement of time-temperature history of a product, a novel strategy has been proposed to use chemical evolution in TTI to correlate with microbial growth in dairy products through a bio-chemo synchronization in a kinetically programmable plasmonic TTI (Figure 4b) [164]. The reaction of epitaxial overgrowth of Ag shell on Au nanorods mimics the bacterial growth in dairy, as both of them are strongly dependent on temperature [169]. By varying the concentrations of reagents, the reaction of chemical evolution is highly synchronized with bacterial growth under different temperatures that may happen during manufacturer-to-consumer chain of each product. Additionally, the chrono-chromic indicator from sharp-contrast multicolor changes during the chemical evolution provides easy readouts for customers. With the indicator on the package undergoing the same temperature history with the milk, customers could directly identify the quality of dairy products.

In contrast to the sensors that are designed to function permanently, biocompatible, eco-friendly, or even edible food sensor becomes increasing attractive for customers. As shown in Figure 4c, an edible passive RFID circuit that use sub-micron thick gold as antennas/resonators and silk as a substrate with good adhesion can be integrated directly onto food such apples or eggs [156]. As a widely used material for over five thousand years, silk emerges as a prime candidate for electronics to interface with living organisms due to its excellent mechanical properties, flexibility, biocompatibility and ease for processing [170,171]. A chemical-free fabrication technique of silk-based sensors has also been developed to avoid contamination from conventional micro-/nano fabrication. Capable of producing silk substrates with various critical feature sizes, the resulting sensor can operate in multiple electromagnetic regimes (e.g., MHz, GHz and THz). When attached to different foods (e.g., egg, apple, cheese and banana), an in-situ monitoring of the change in resonant responses indicates the spoilage process.

When all components of the sensor on the food are edible and biodegradable, people will not need to remove them before eating the food. In order to achieve stable operation before function degradation, encapsulation strategy has to be specifically explored. Such a sensor would allow monitoring of the signal in situ over the course of digestion with the sensor traveling in the body. A recent effort to design totally edible electrochemical electrodes has explored food materials, including edible food sleeves, olive oil and edible activated charcoal (Figure 4d) [165]. Packed inside food (e.g., hollow penne, cookie and green bean), these electrodes are capable of well-defined voltammetric measurements of uric acid, ascorbic acid and dopamine. In the voltammetric measurement, the voltammagram plots the current produced by the analyte as a function of the potential of the working electrode [172].

Additionally, the demonstrations in simulated saliva, gastric fluid and intestinal fluid demonstrate their effectiveness to take sensitive measurements. Common graphite-powder/mineral-oil based carbon paste electrodes (CPEs) are similar in performance and conductivity compared to the edible CPEs. Being completely dissolvable inside the human body, this new type of sensors provides access to biomarkers that were previously inaccessible for real time monitoring. Due to increasing interest and popularity in wearable or even edible sensors applied in the healthcare, the security and privacy concerns should be addressed. In November 2017, the first digital pill was approved by the FDA [173]. Integrated with a tiny ingestible sensor, the digital pill allows transmission of medication data to a smartphone app. The patient can also voluntarily upload the collected data to an online database, with access given to the relevant doctor and other authorized personnel. It is critically important to address the concern on data security, before commercialization and wide adoption of the product.

### 4.2. Application in Zoology

**Figure 5:**
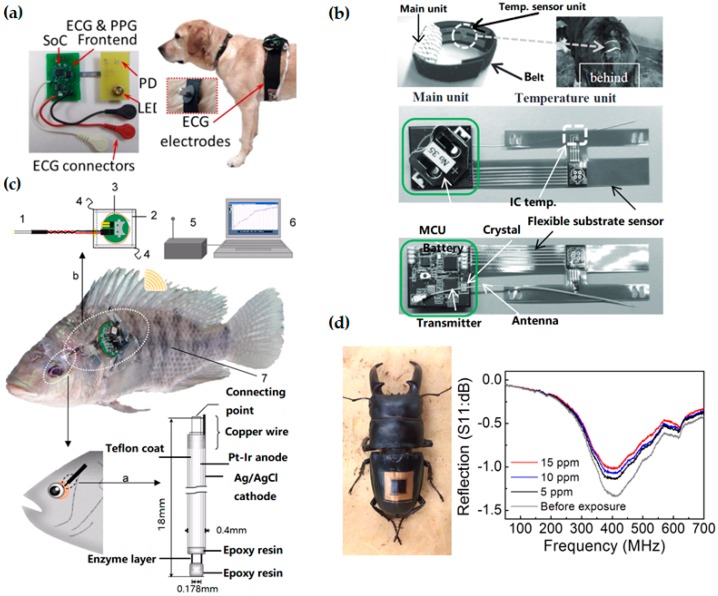
Wearable technologies in animal care. (**a**) Printed circuit boards for electrocardiography (ECG) and photoplethysmogram (PPG) measurements and Bluetooth wireless transmission (**left**), integrated on the chest of a dog (**right**). Reprinted with permissions from [181]. (**b**) A flexible temperature sensing unit with another unit to process and transmit the data. Reprinted with permissions from [182]. (**c**) Schematic diagram of the wireless monitoring system for fish: (1) needle-type enzyme sensor; (2) waterproof sheet; (3) wireless potentiostat; (4) nylon threads; (5) receiver; (6) personal computer; and (7) sample fish (Nile tilapia). Adapted with permissions from [183]. (**d**) Photograph of a wireless sensor integrated onto the epidermis of the insect for monitoring of dimethyl methylphosphonate (DMMP) vapor. Scale bar, 1 cm. Reprinted with permissions from [73].

Wearable biosensors also contribute to in situ monitoring of animals and plants, with applications that include disease detection of livestock [174], estimating water status of leaves [175] and many others [176,177,178]. As dogs perform many roles for people (e.g., hunting, herding, assisting police and military, companionship and aiding handicapped individuals), wearable sensors have been used on canines to monitor their health status for improving human-canine interactions. Compared to traditional implantable sensors, a non-invasive wireless system with electrocardiography (ECG) and photoplethysmogram (PPG) sensors on a chest strap (Figure 5a) can monitor a canine’s physiology (heart rate, heart rate variability and respiration rate) during different behaviors (sitting, lying down, standing) [179]. The thick canine fur layer is a main barrier for accurate measurement of both ECG and PPG signals. To address this challenge, pointed style stainless steel electrodes have been used for ECG and efficient optical coupling light pipes to the skin has been designed for PPG. Taken together with accelerometers and gyroscopes [180], the set of data transmitted wirelessly to a computational node could be analyzed to inform the dog’s behavior and physiological state for canine training and other human-canine interactions. It is worth mentioning that the humanity issue should be considered when working with animals.

Instead of relying just on farmers’ senses and experiences, the use of biosensors and wearable technologies for livestock can provide reliable data about the status of the animals, allowing animal owners to run their farms in a more efficient and profitable way [184]. For example, estrous detection is one of the most important factors for improving reproductive performance in the dairy and beef cattle industries [185]. Estrous detection has so far been limited by the poor detection accuracy or high testing cost of various estrus detection tools (EDT) [186,187,188]. Because of the strong correlation between body temperature and estrus in cattle [188,189,190], continuous in situ monitoring of the body temperature throughout the estrous cycle could be effective. Figure 5b presents a flexible temperature sensing unit with another unit to process and transmit the data [182]. Easily appressed to the ventral base of a calf’s tail (one of the slimmest parts), the less stressful and invasive system could investigate changes in surface temperature onset of estrus and ovulation throughout the estrous cycle of cattle. Additionally, in situ surface temperature monitoring could help detect many diseases in cattle such as respiratory disease, the number-one cause of death in calves [191].

In face of changing circumstance, animal may also feel “stressed” as humans do, which would affect their health status. The stress of fish from alterations of water chemistry (e.g., dissolved oxygen, pH and etc.) and behavioral interactions between individuals (including attacking behavior and visual irritation) has been investigated [192]. As a representative of the respiratory and nutritional state, glucose level has been demonstrated to closely correlate to the stress level in fish [193]. Consisting of a needle-type amperometric biosensor, a wireless potentiostat to apply the working potential, a receiver and a personal computer (Figure 5c), the wearable monitoring system inserted into the eyeball sclera can detect the glucose concentration in the eyeball interstitial sclera (EISF) of fish [183]. In such underwater application, a waterproof design has been developed, with the potentiostat covered by a waterproof polypropylene sheet and sealed by a thermo-compression bonding device. Attached to the dorsal and pectoral fins of the fish by using nylon thread, the waterproofed wireless potentiostat can communicate wirelessly to a receiver at a radio frequency of 916.5 MHz. Because of insufficient battery power in the current study, the monitoring from the wireless potentiostat could not continue for more than 3 days. This system could provide biologist a convenient method for real-time monitoring of glucose levels in fish under free-swimming conditions in an aquarium. A similar concept has been applied for real-time monitoring of lactate [194] and cholesterol in fish [195], which are important indicators for the stress and physiological status of fish. In addition to the study of fish, the real-time monitoring of fish also helps fishery owner to detect abnormalities of fish for increased productivity.

### 4.3. Application in Botany

**Figure 6:**
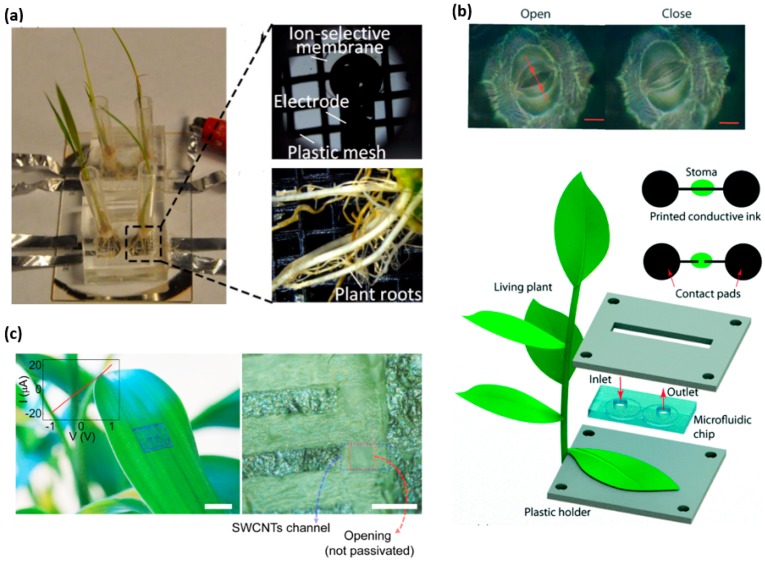
Wearable sensors for the plant study. (**a**) Photos of the fabricated ion-selective electrode and plant roots inset the growth chamber. Reprinted with permissions from [197]. (**b**) A microfluidic channel to deposit conducting ink across an open stoma, creating an electronic circuit that is intact. When the stoma pore is closed, the current could be measured. Reprinted with permissions from [200]. (**c**) A photograph (**Left**) an optical microscope image (**Right**) and the *I–V* characteristic (inset) of graphite arrays transferred onto surface of a live leaf. Reprinted with permissions from [73].

In the field of agriculture and botany, there is an established demand for the real-time monitoring of biomarkers in plants. One specific focus is to monitor the nutrient uptake rate in real time to understand the health and growth rate of the plant. The essential nutrients needed in larger quantities contain elements such as C, H, O, N, P, K, S, Ca and Mg [196]. By using ion-selective electrodes, an in situ microfluidic plant sensor can monitor nitrate and phosphorous uptake in real time [197]. In the experimental setup, the plant roots are placed on the sensor that contains a growth medium with ingredients of known concentrations. The plant feeds upon these nutrients, causing a decrease of concentration in the medium. A potentiometric sensor provides detection of the concentration of the nutrients. Two Ag/AgCl ion-selective electrodes in the sensor form a working electrode and reference electrode pair to measure the electromotive force (EMF) between the pair [198]. The decrease in the concentration of the nutrients present in the growth medium causes a decrease in the EMF, which is quantified and recorded by an external electrochemical workstation. This sensing apparatus as shown in Figure 6a quantitatively monitor the H_2_PO_4_^−^ and NO_3_^−^ intake rate over the span of two weeks with fast response rate, high ion selectivity and low cost installation [199].

Water availability is a major limiting factor for plant growth. When soil dries out, plants reduce photosynthetic activity and suffer tissue damage [201]. As the earliest and the fastest drought resistance mechanism, plants regulate their transpiration rates through stomata-small pores (5–10 μm) in the surface of a leaf [202]. Considering stomatal aperture as a promising indicator, a stomatal electro-mechanical pore size sensor (SEMPSS) has been developed to trace single stoma-aperture dynamics for persistent drought monitoring of plants [200]. To avoid damaging the stomata, water-based ink with carbon nanotubes and sodium dodecyl sulfate as a surfactant has been employed as the conducting ink. With a microfluidic channel to deposit the conducting ink across an open stoma, the created electronic circuit allows the measurement of the current when the stoma pore is closed and vice versa (Figure 6b). Therefore, the stomatal aperture can be monitored in real time via electrical resistance measurements. Such a capability provides stomatal opening and closing latencies of plants under different environmental conditions, which are important yet previously inaccessible for monitoring of plant states. This biocompatible printing of conductive circuits directly onto the leaf with a micrometer precision may enable more complex electronic functions (e.g., coupled radio-frequency identification, electrochemical and logic circuits), enabling the monitoring and engineering of novel plant functions.

Integrating new materials into the design of wearable sensors could open up new capabilities and potential applications. Due to high carrier mobility, high electrical and thermal conductivity and mechanical flexibility [203], carbon nanostructures enable a new type of in situ sensors based on single-walled carbon nanotube (SWCNT) channels and graphitic electrodes [73]. Because of great adhesion and mechanical flexibility [204], the sensor can be transferred to non-planar biological surfaces without affecting its operation. Furthermore, the sensor network can be interfaced with inherent life forms in nature for monitoring environmental condition. As shown in Figure 5d and Figure 6c, the SWCNTs–graphite sensor is integrated on a live insect and a live plant for real-time, wireless sensing of toxic gases. The performance of the sensor has been evaluated for monitoring environmental conditions by exposing the sensor to a sarin nerve agent in different air humidity and concentrations. The sensor can also monitor the pollutions and infections present in the environment. The inlaid graph in Figure 6c depicts the linear current-voltage (I–V) characteristics, indicating an Ohmic contact between the carbon nanotube channels and graphitic electrodes in the array of field-effect transistors even on the leaf surface. However, the noise occurs during measurement partially due to humidity change and imperfect contact between graphitic interconnect pads and the tips of the measurement probe.

## 5. Future Perspectives and Conclusions

### 5.1. Future Perspectives

Among a variety of wearable sensors, fitness tracking devices become popular and beneficial for fitness professionals and the general public for personal healthcare [205]. Compared to these commercial sensors/devices, non-invasive biosensors still remain a distant prospect [5]. Considering the small sampling volumes collected by wearable biosensors and low bioanalyte concentration in these samples, it is of growing interest to pursue biosensors with excellent selectivity, sensitivity and reproducibility. Long-term biocompatibility and mechanical robustness of wearable biosensors also need to be addressed before the commercialization. Most of the demonstrated wearable chemical sensors focused on monitoring of a single analyte. Integration of different sensing modalities into a single, multifunctional platform is of particular interest for improved sensing accuracy. Due to the large footprint of the data/energy transmission components, the high-density integration could be challenging. The development of miniaturized power source, as well as data transmission component, represents an important direction for future research. As a promising example, biofuel cells that are capable of harvesting energy from biofluids through local oxidation can potentially serve as a long-term power source for wearable biosensors [206]. In addition to the technological advancement, the cost and appearance associated with the sensor are important for the successful commercialization as well [207]. Additionally, there is a parallel development in wearable sensors, pertaining the usage of degradable materials. Without toxic chemicals, these degradable sensors aim to disappear without a trace in either the environment or the human body, removing the need for recollection. Taken together with the concept of edible food sensors, the biodegradable characteristics of the material may open a new frontier for bioanalyte analysis.

### 5.2. Conclusions

In this mini review, we have briefly discussed the background of bioanalyte monitoring and introduced the recent development of wearable biosensing technologies. Combined with wearable technology, novel non-invasive biosensors provide continuous in situ monitoring capabilities that could not be achieved by traditional methods. Such wearable monitoring devices have been widely applied in diverse fields, ranging from human and animal health monitoring to tracking of food quality and plants status. Carefully addressing the critical roadblocks on the path of commercialization would bring this exciting technology into fruition, opening up opportunities for an even broader range of applications.

## Figures and Tables

**Table 1:** Summary of wearable biosensors.

Platform	Biofluid	Measuring Method	Analyte	Sensitivity	Range of Detection	Power Source	Reference
Nose-bridge pads of glasses	Sweat	Amperometry *^1^, Potentiometry *^2^	Potassium, lactate, glucose	Lactate:	Lactate: 0.1 to 14 mM	PCBs with Li-ion battery	[104]
				0.8 μA/mM	potassium: 0.1 to 100 mM		
				potassium:	glucose: N/A		
				60.6 mV/M			
				glucose:			
				2 μA/mM			
Wearable FISA	Sweat	Potentiometry, Amperometry, Commercial temperature sensor	Glucose, lactate, sodium, potassium, skin temperature	Glucose: 64.2 mV/dec Potassium: 61.3 mV/dec Temperature: 0.18%/°C	Glucose:	PCBs with Li-ion battery	[128]
					0 to 200 μM		
					lactate:		
					2 to 30 mM.		
					potassium:		
					2 to 16 mM		
					sodium:		
					20 to 120 mM		
Epidermal microfluidic patch	Sweat	Colorimetric analysis	pH, sweat rate, lactate, glucose, chloride,	pH: 0.5/%1 R	pH: 5.0 to 7.0	NA	[98]
				glucose:	glucose:		
				0.1 mM/%1 R	1.5 to 25mM		
				lactate:	lactate:		
				0.3 mM/%1 R	1.5 to 100 mM		
				chloride:	chloride:		
				0.2 mM/%1 R	39 to 625 mM		
Tattoo-like biosensor	ISF	Colorimetric and fluorescent analysis	pH, Sodium, glucose,	--	pH: 7.0 to 9.0	NA	[129]
					sodium:		
					25 to 100 mmol/L		
					glucose:		
					5 to 110 mmol/L		
Mouthguard	Saliva	Amperometry	Uric acid	1.08 µA/mM	50 µM to 1 mM	Watch battery	[130]
Contact lens	Tear	Field-effect transistor, passive RLC circuit	Glucose, intraocular pressure	Intraocular	Glucose: 0.1 to 0.6 mM	Reader antenna	[131]
				Pressure: 2.64 MHz mm Hg^−1^	intraocular pressure:		
					5 to 50 mm Hg		
E-nose	Armpit odor	Conducting polymer	VOCs (ammonia, acetic acid, acetone, ethanol)	--	--	Battery for comm. device	[107]

*^1^ Amperometric sensors: devices that measure the current produced during the oxidation or reduction of electroactive species at a constant applied potential. This current is proportional to the concentration of the electroactive product. *^2^ Potentiometric sensors: devices that measure the electromotive force generated between two electrodes. The measured electromotive force has a direct dependence on the analyte concentration.
